# Beneficial effects of pioglitazone and metformin in murine model of polycystic ovaries via improvement of chemerin gene up-regulation

**DOI:** 10.1186/2008-2231-22-39

**Published:** 2014-04-24

**Authors:** Nahid Kabiri, Mohammad Reza Tabandeh, Seyed Reza Fatemi Tabatabaie

**Affiliations:** 1 Department of Physiology, Faculty of Veterinary Medicine, Shahid Chamran University of Ahvaz, Ahvaz, Iran; 2 Department of Biochemistry and Molecular Biology, Faculty of Veterinary Medicine, Shahid Chamran University of Ahvaz, Ahvaz, Iran

**Keywords:** PCO, Chemerin, Gene expression, Ovary, Pioglitazone, Metformin

## Abstract

**Background:**

Polycystic ovary syndrome (PCO) is recognized as the most common endocrinopathy in female. Chemerin is a novel adipocytokine that is expressed in ovary and upregulated in adipose tissue of obese, PCO patients. To date there is no report about the regulation of ovarian chemerin gene expression after PCO induction and treatment by insulin sensitizing drugs including pioglitazone and metformin.

Thirty female rats were divided into six experimental groups with five rats in each group including control group, PCO group (i.m injection of 4 mg estradiol benzoate for 40 days), metformin treated (200 mg/kg/day for 21 days), pioglitazone treated (20 mg/kg/day, for 21 days), PCO + metformin and PCO + pioglitazone. PCO was detected by microscopic observation of vaginal smear and treatment by metformin and pioglitazone was initiated one week after that. Ovarian chemerin expression was analyzed by real time PCR and western blotting.

**Results:**

Our results demonstrated that PCO induction resulted in elevation of chemerin mRNA and protein levels in ovary in concomitant with incidence of insulin resistance and increasing androgen and progesterone production. We observed that metformin and pioglitazone attenuated ovarian chemerin expression and improved insulin resistance and abnormal steroid production in PCO rats.

**Conclusion:**

Based on data presented here we concluded that alteration of ovarian chemerin expression may has important role in PCO development and manipulation of chemerin expression or signaling by pioglitazone or metformin can be a novel therapeutic mechanism in the treatment of PCO patients by these drugs.

## Background

Polycystic ovary syndrome (PCO) is recognized as the most common endocrinopathy in female during the reproductive age. PCO is characterized by reproductive dysfunction symptoms such as hyperandrogenic chronic anovulation due to excess androgen production, menstrual disturbances, infertility or subfertility and the presence of enlarged, sclerocystic ovaries. Obesity, insulin resistance, pancreatic-cell dysfunction and impaired glucose tolerance occur at least in 50% of women with PCO [1,2]. Previous reports have shown that hyperinsulinemia in these patients dysregulates LH secretion and promotes ovarian androgen secretion resulting in elevation of free androgen level [3].

Over the past decade it has been shown that adipocytes are secretory cells that produce a variety of proteins with hormonal actions, which collectively have been called adipocytokines. These cytokines have important roles in male and female reproductive physiology by regulation of carbohydrate and lipid metabolism in adipose or reproductive tissues such as hypothalamus-pituitary axis, ovary, uterus and embryo [4,5]. Among them, adiponectin, leptin, resistin and visfatin are the major adipocytokines which their changes in serum or adipose tissue have been identified in patients with obesity, insulin resistance and PCO [6,7]. Various secretion or gene expression patterns of adipocytokines or their receptors have been detected in adipose tissue or ovary of PCO patients [8,9].

Chemerin is a novel adipocytokine which is predominantly expressed by adipocytes and its gene expression elevates in the adipose tissue of obese animals and human [7]. High correlations have been found between plasma chemerin level and different features of metabolic syndrome including body mass index, plasma triglycerides and blood pressure in human [10]. Chemerin knockdown animals demonstrate impaired adipocyte gene expression and unregulated glucose and carbohydrate metabolism [11]. Recent data demonstrate that recombinant chemerin promotes angiogenesis; a mechanism which is essential for adipose tissue expansion in obese patients [11,12]. Recently, inhibitory action of chemerin on FSH or IGF-1 induced steroidogenesis have been reported in granulosa cells [13-15]. Serum chemerin increases in women with PCO [16].

Insulin-sensitizing drugs (ISDs) such as metformin or thiazolidinediones (TZDs) ameliorate reproductive abnormalities, restore ovulation and regular estrous cycle, increase pregnancy rates and reduce androgenic symptoms in women with PCO [17,18]. The positive actions of these insulin-sensitizing agents on adipocytokine secretion or expression in adipose tissue or ovaries of PCO patients have been described. However the full mechanism of action of these two drugs in ovaries of PCO patient is still unraveled and further investigations remain to elucidate the precise mechanism of actions of these drugs at molecular levels.

To our knowledge, no study is available that identify the changes of chemerin gene expression in ovaries of animals or human with PCO and that compare the effects of pioglitazone and metformin on its gene expression in experimental model of PCO. Here for the first time we showed that ovarian chemerin gene expression changed after PCO induction and pioglitazone or metformin treatment.

## Methods

### Rat treatment regimes

Thirty Female Sprague-Dawley rats (3 month of age) weighing 150-180 g were housed in a temperature-controlled room (23 ± 1°C) with a 12 h light/dark cycle and were provided rat chow (Pars, Tehran, Iran) and water at libitum.

All animals used were cared for according to the Guide for the Care and Use of Laboratory Animals by the National Academy of Sciences (National Institutes of Health publication No. 86-23). The rats were allowed to acclimatize for 10 days before the beginning of the experiment. The estrous cyclicity was monitored by vaginal smears obtained between 08:00 and 12:00 hours and the rats with abnormal cyclicity were dismissed from the experiment.

The rats were divided into six experimental groups with five rats in each group: 1) healthy control (vehicle control), 2) PCO, 3) metformin treated 4) pioglitazone treated, 5) PCO and metformin treated and 6) PCO and pioglitazone treated. PCO was induced by a single i.m injection of 4 mg estradiol valerate (Loghman Pharmaceutical & Hygienic Co, Karaj, Iran) in 0.2 ml sesame oil (Barij Essense, Kashan, Iran) for 40 days [19] and detected by microscopic observation of vaginal smear. The presence of prolonged cornified cells for at least two consecutive estrous cycles was used as successful PCO induction. Treatment with metformin and pioglitazone was initiated one week after PCO induction.

Animals in metformin treated groups (groups 3 and 5) were received 200 mg/kg/day metformin (Loghman Pharmaceutical & Hygienic Co, Karaj, Iran) by gavage method for 21 days. Pioglitazone (Dorsa Pharmaceutical Co. Tehran, Iran) was daily used for the 21 days duration as the same method as metformin groups with dose of 20 mg/kg/day. Dosages of pioglitazone and metformin were chosen based on dosages that were clinically used in human patients (25-30 mg/kg/day for pioglitazone and 200-300 mg/kg/day for metformin).

### Tissue and serum sampling

Animals were anesthetized by chloroform and serum samples were obtained by cardiac puncture, separated by centrifuging at 5,000 rpm for 5 minutes and stored at -20°C for the subsequent assays. Both ovaries were carefully trimmed of adhering fat and connective tissue, pooled and immediately frozen in liquid nitrogen at -80°C. The body weight and length (nose to anus lenght) of all rats were determined at the end of study and body mass index (BMI) was calculated by using following formula as described previously; body weight (g)/Length^2^ (cm^2^) [20]. Control ovaries were collected from rats at estrous or proestrous stages of cycle.

### Ovarian morphology

Some ovary was removed, cleaned of adherent connective fat tissue, and fixed in 4% formaldehyde buffer for at least 24 hours. Thereafter the samples were dehydrated and imbedded in paraffin. The ovaries were partially longitudinally sectioned (4 μm, every tenth section mounted on the glass slide), stained with hematoxylin and eosin and the presence of healthy and atretic follicles, follicular cysts and corpora lutea was assayed. Antral follicles with pyknotic granulosa and theca layers, unhealthy, degenerative oocyte and filled in antral cavity with numerous apoptotic derbies were characterized as atretic follicles. Preantral follicles with degenerative oocyte and pyknotic granulosa layer were assigned as atretic follicles.

### Hormone and glucose assays

Serum insulin levels were measured by using the Rat ELISA kits (Mercodia, Sweden) according to the manufacturer’s recommendation. Insulin concentration was expressed as μg/L. The limit of detection of insulin was 0.01 μg/L and the intra-assay and interassay coefficients of variation were less than 4% and 8.13%, respectively. The calibrator ranges were between 0-10 μg/L (first calibrator at 0.01 μg/L).

The progesterone (P4) and testosterone (T) concentrations were determined by using a commercial radioimmunoassay kit (Immunotech, Radiová, Czech Republic). The limit of detection of P4 was 0.1 ng/ml, and the intra-assay and interassay coefficients of variation were less than 10% and 11%, respectively. The calibrator ranges were between 0 - 100 ng/mL (first calibrator at 0.1 ng/mL). The limit of detection of T was 0.1 ng/ml, and the intra-assay and interassay coefficients of variation were less than 12.1% and 11.2%, respectively. The calibrator ranges were between 0 – 25 ng/mL (first calibrator at 0.1 ng/mL). Blood glucose was determined by glucose oxidase method (Pars Azmoon, Tehran, Iran) as described by manufacturer.

### HOMA-IR estimation

HOMA-IR was estimated through previously described formula (fasting glucose × fasting insulin/22.5) and represented by unit of mmol/L × μU/ml [21].

### RNA preparation

Total RNA was extracted from ovaries using TriPure total RNA isolation kit according to the manufacturer’s procedure (Roche Molecular System, USA), dissolved in dimethyl pyrocarbonate treated water and quantified at a wavelength of 260 nm by nanodrop spectrophotometry (Eppendorf, Hamburg, Germany). The RNA with optical density absorption ratio OD260 nm/OD280 nm between 1.8 and 2.0 was used for reverse transcription (RT) reaction. Genomic DNA was removed by treating 1 μg of isolated RNA with 2 units of DNase I (Fermentas Inc, Vilnius, Lithuania).

### Reverse transcription − polymerase chain reaction

Reverse transcription was done in a total volume of 20-μl by using an AmpliSence cDNA synthesis kit (AmpliSens Enterovirus-Eph, Russia) as recommended by the manufacturer. The PCR reactions was performed in a 25-μl reaction using Taq DNA polymerase (Cinagen Co, Iran) and a thermal cycler (Eppendorf Mastercycler, Hamburg, Germany). Specific sets of primers (BIONEER, Seoul, South Korea) that used for amplification of rat chemerin were as follows; forward: 5′-ATGGCGGGCAACGGCGCCAT-3′ and reverse: 5′-CCATCAACGTCGTCAACTAA-3′. Thermal conditions for amplification of chemerin were 35 cycles consisting of denaturing at 94°C for 1 min, annealing at 58°C for 1 min, extension at 72°C for 1 min, with an initial denaturing step at 95°C for 10 min and a final extension step at 72°C for 10 min. cDNA from adipose tissue was used as positive control. Expression of chemerin in rat ovaries was confirmed by visualization of PCR product on agarose gel electrophoresis (1%).

### Real time PCR

To evaluate the levels of chemerin gene expression in ovaries of different animals quantitative real-time PCR (qRT-PCR) was performed using the ABI Step One plus real-time PCR detection system (ABI plus; Applied Biosystems, USA), and qPCR™ Green master kit for SYBR Green I® (Applied Biosystems, USA). Relative expression level of chemerin transcript was compared to GAPDH as housekeeping gene. Specific sets of primers (Macrogen, Seoul, South Korea) that were used for amplification of rat GAPDH [GenBank: NM_017008.4] and chemerin [GenBank: NM_001013427] genes were designed using Beacon Designer 7.1. Sequences of primers for amplification of rat GAPDH and chemerin (BIONEER, Seoul, South Korea) were as follows; rat chemerin: 5′-TGTGGACAGTGCTGATGACCTGTT-3′ and 5′-CAGTTTGATGCAGGCCAGGCATTT-3′ and rat GAPDH: 5′-CTCATCTACCTCTCCATCGTCTG-3′ and 5′-CCTGCTCTTGTCTGCCGGTGCTTG-3′ Real time PCR reactions were performed with the following settings: 5 minutes of pre-incubation at 95°C followed by 40 cycles for 15 seconds at 95°C and 45 second at 60°C. Reactions were performed in triplicate. A reaction without cDNA was performed in parallel as negative control.

Relative quantification was performed according to the comparative 2^-ΔΔCt^. For analysis of qRT-PCR results based on ΔΔCt method StepOne™ software was used. The result for the gene expression was given by a unitless value through the formula 2^-ΔΔCt^. Validation of assay to check that the primer for the GAPDH and chemerin had similar amplification efficiencies was performed as described previously [22].

### Western blotting

Total protein from isolated ovaries was precipitated after RNA and DNA isolation using TriPure total RNA isolation kit according to the manufacturer’s procedure (Roche Molecular System, USA) and its concentration was measured using Bradford method as described previously [23]. Twenty five μl of each protein sample (1 μg/μl) were mixed with 25 μl Laemmli sample buffer supplemented with 2-mercaptoethanol at a final concentration of 7.5% (vol/vol). The samples were heated for 15 min at 65°C, separated by 10% SDS-PAGE and electrophoretically transferred to a nitrocellulose membrane (Schleicher & Schuell, Inc., Keene, NH). The filters were blocked by incubation for 1 h in PBS with 5% nonfat milk. Blots were then washed in PBS-Tween and immunoblotted with primary antibody against mouse chemerin (Abcam, Cambridge, UK, Art No: ab112450) at 1:500 ratio. Detection of primary antibody was done using goat anti rabbit HRP-conjugated antibody (Abcam, Cambridge, UK, Art No; ab98467) at 1:1000 ratio and DAB reagent (Sigma Aldrich, Germany). Densitometric quantification of chemerin proteins in relation to GAPDH as calibrator was performed using Image J software (National Institutes of Health). Western blot was done in three independent experiments for each sample.

### Statistical analyses

Data analyses were done using the SPSS 16.0 software package (SPSS Inc., Chicago, IL, USA). Two-way analysis of variance (ANOVA) and general linear model was fit to evaluate the effect of PCO status (PCO vs. control) and treatments (treatment with metformin or pioglitazon vs. no treatment) on each variable. The two-way ANOVA model is given by Yijk = μ + α_i_ + β_j_ + (αβ)_ij_, where α_i_ and β_j_ represent the effects of PCO induction and treatment, and (αβ)_ij_ represents the interaction of two factors. The Spearman rank correlation coefficient was used to estimate the correlation between chemerin gene expression and different parameters. All experimental data were presented as the mean ± SD. The level of significance for all tests was set at P < 0.05.

## Result

**Figure 1:**
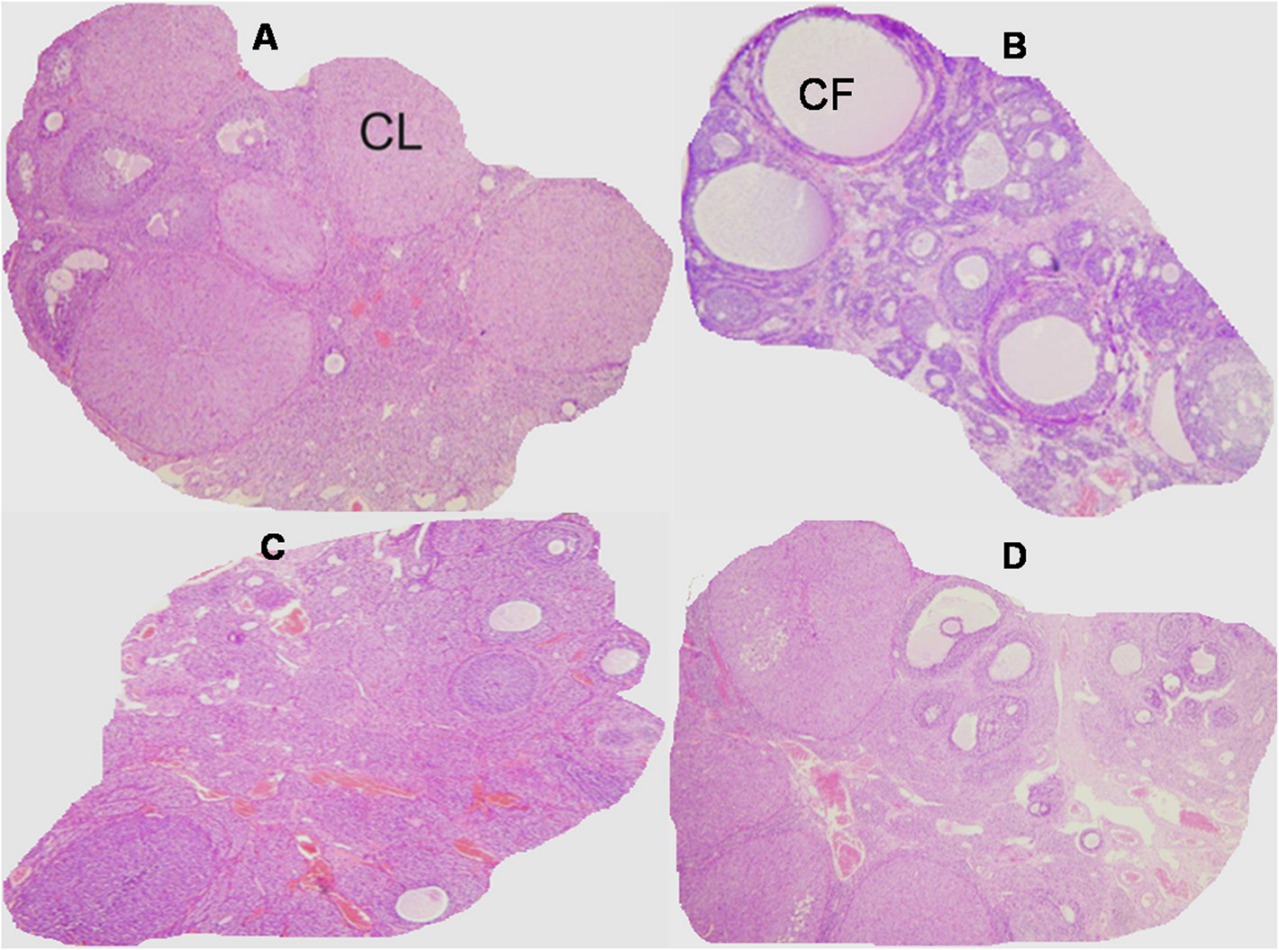
**Ovarian features in normal rats (A)**, **estradiol induced PCO rats**, **(B) PCO rats treated with pioglitazone (C) and PCO rats treated with metformin (D).** Normal rats displayed follicles at different stages and the presence of corpora lutea (CL) **(A)**, while estradiol treatment resulted in formation of higher numbers of atretic and cystic follicles (CF) and the absence of CL **(B)**. Metformin and pioglitazone treated rats **(C and D)** exhibited lower numbers of CF, higher numbers of antral follicles and growing young CL. H & E staining (magnification × 40).

The ovaries in the control group exhibited a typically normal appearance with small and medium sized antral follicles and corpora lutea (Figure 1A). The ovaries in the PCO group displayed typical PCO-like changes including presence of higher numbers of atretic and cystic follicles compared with the control group (Figure 1B). Metformin and pioglitazone treated rats exhibited lower numbers of atretic and cystic follicles, higher numbers of antral follicles and growing young corpora lutea compared with PCO group (Figure 1C-D). Restoration of normal follicular appearance was more pronounced in metformin treated rats compared with pioglitazon treated animals (Figure 1C-D).

**Figure 2:**
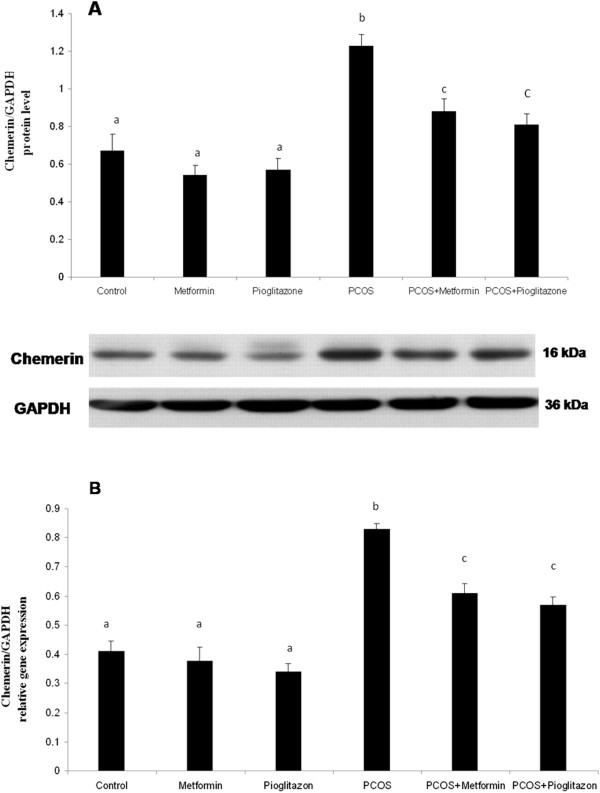
**Chemerin protein (A) and mRNA levels (B) in rat ovaries after PCO induction and treatment with pioglitazone and metformin (n** = **5 in each group).** Chemerin was up-regulated in ovary of PCO rats compared with control animals, whereas its level decreased after treatment with metformin and pioglitazone. Data were presented as the mean ± SD. Different letters denote differences among groups at P < 0.05.

The real time-PCR and western blot results demonstrated that chemerin mRNA and protein levels were elevated in ovaries of PCO rats when compared to that in healthy animals (*P* < *0.05*) (Figure 2). The chemerin gene expression levels were decreased in ovaries of PCO rats in response to treatment with metformin and pioglitazone compared to untreated PCO rats (*p* < 0.05) (Figure 2). Metformin and pioglitazone had no effects on the levels of chemerin mRNA and protein in healthy treated rats in relation to healthy untreated animals regardless of PCO status (Figure 2) (*p* > *0.05*).

**Figure 3:**
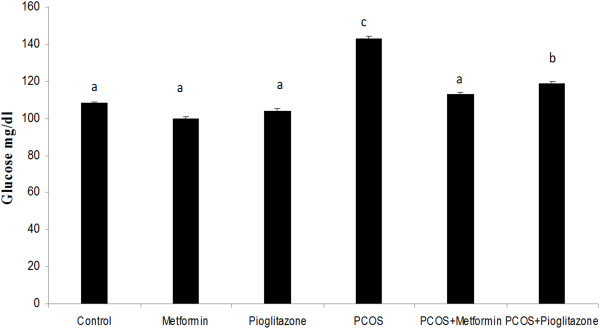
**Serum glucose levels (mg/dl) in normal rats (n = 5), PCO induced rats (n = 5) and treated rats with metformin and pioglitazone (n = 5).** Serum glucose was elevated in PCO rats compared with control animals. Treatment of PCO rats with metformin and pioglitazone resulted in reduction of glucose level in relation to PCO untreated rats. Data were presented as the mean ± SD. Different letters denote differences among groups at P < 0.05.

**Figure 4:**
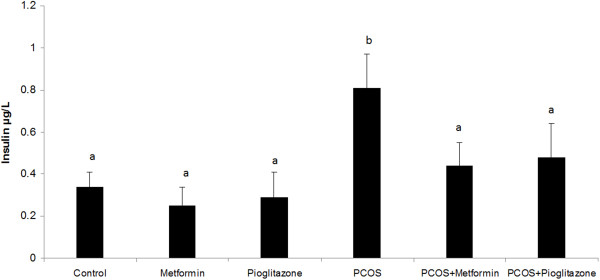
**Serum insulin levels (ug/L) in normal rats (n = 5), PCO induced rats (n = 5) and treated rats with metformin and pioglitazone (n = 5).** Serum insulin level was considerably higher in PCO rats compared with that in normal animals. Treatment of PCO rats with metformin and pioglitazone decreased the over-secretion of insulin in relation to PCO untreated rats. Data were presented as the mean ± SD. Different letters denote differences among groups at P < 0.05.

As shown in Figures 3 and 4 plasma glucose and insulin levels were higher in the PCO induced rats (1.92 ± 0.38 fold) when compared with normal untreated group (*p* <0.05) after controlling for the effect of treatment. Treatment of PCO rats with metformin and pioglitazone resulted in reduction of glucose (1.36 ± 0.27) and insulin (1.82 ± 0.36) levels in relation to PCO untreated rats (*p* < 0.05) (Figures 3 and 4). In healthy treated groups plasma insulin and glucose levels had no differences with healthy untreated animals (Figures 3 and 4), (*p* > *0.05*) after controlling for PCO status.

**Figure 5:**
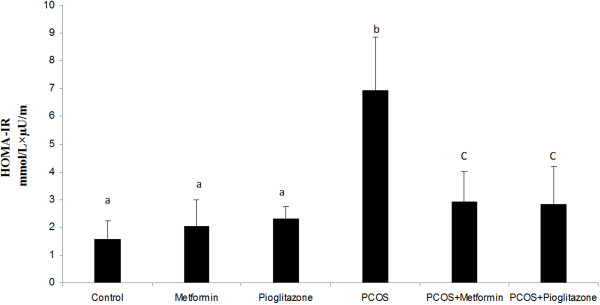
**HOMA**-**IR index (mmol/L × uU/ml) in normal rats (n = 5), PCO induced rats (n = 5) and treated rats with metformin and pioglitazone (n = 5).** Rats with PCO showed insulin resistance compared with normal rats, while treatment of those with metformin and pioglitazone attenuate insulin resistance in relation to PCO untreated rats. Data were presented as the mean ± SD. Different letters denote differences among groups at P < 0.05.

The HOMA-IR index, which reflects whole body insulin resistance, was increased in PCO animals (3.63 ± 0.41 fold) (*p* < 0.05) (Figure 5) regardless of treatments. Metformin and pioglitazone induced reduction of HOMA-IR in PCO rats about 2.8 fold compared with untreated PCO animals (*p* < 0.05) (Figure 5). Healthy treated rats did not show clear changes in HOMA-IR level in relation to healthy untreated rats after controlling PCO status.

**Figure 6:**
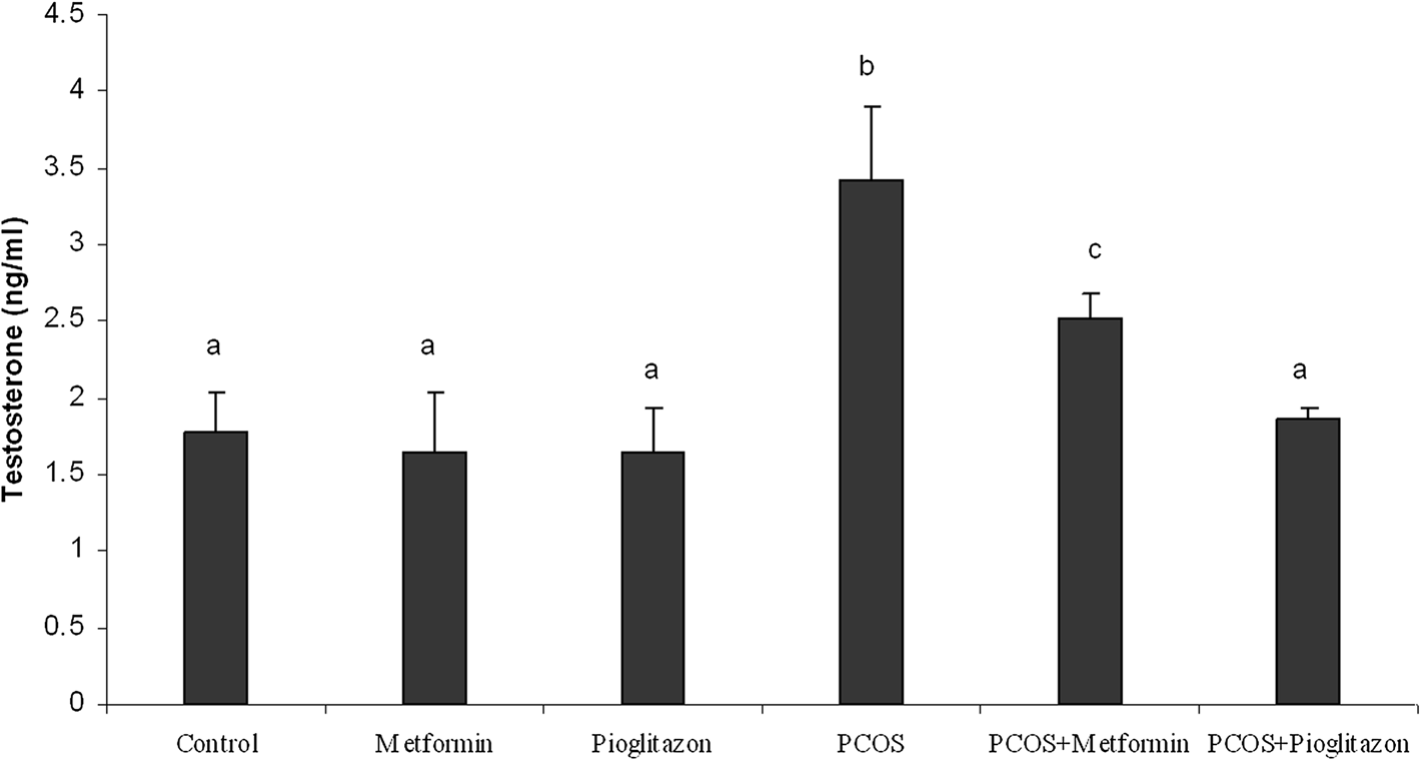
**Serum testosterone (T) levels (ug/L) in normal rats (n = 5), PCO induced rats (n = 5) and treated rats with metformin and pioglitazone (n = 5).** Induction of PCO caused an increasing the T level, while metformin and pioglitazone attenuated over secretion of T. Data were presented as the mean ± SD. Different letters denote differences among groups at P < 0.05.

**Figure 7:**
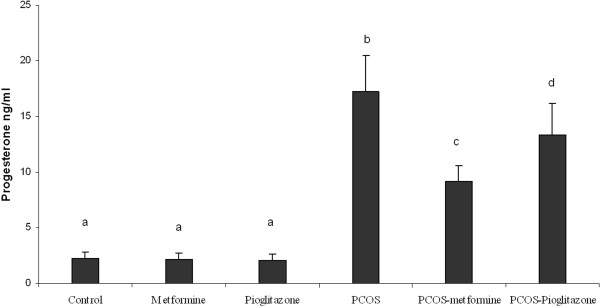
**Serum progesterone (P) levels (ug/L) in normal rats (n = 5), PCO induced rats (n = 5) and treated rats with metformin and pioglitazone (n = 5).** Induction of PCO caused an increasing the P level, while metformin and pioglitazone attenuated over secretion of T. Data were presented as the mean ± SD. Different letters denote differences among groups at P < 0.05.

The plasma concentrations of T and P4 are shown in Figures 6 and 7 respectively. We found a 1.8 ± 0.3 fold increase of plasma T and a 6.9 ± 0.8 fold increase of P4 rats after induction of PCO (*p* < 0.05) (Figures 6 and 7) regardless of treatments. We found that in treated PCO rats, T concentrations were decreased by 1.2 ± 0.3 fold and 1.9 ± 0.5 fold after metformin and pioglitazone treatment respectively (*p* < 0.05) (Figure 6). The reduction of T level was more pronounced in PCO rats in the presence of pioglitazone compared with metformin (*p* < 0.05) (Figure 6), while metformin was more effective in reducing P4 concentration in this group (*p* < 0.05) (Figure 7).

Spearman Rank correlation analyses demonstrated that ovarian chemerin gene expression was positively associated with P4 (*p* < 0.01), HOMA-IR, BMI, insulin and glucose levels (*p* < 0.05) (Table 1). Δ chemerin gene expression in treated PCO animals had positive correlation with Δ insulin, Δ HOMA-IR and Δ P4 (*p* < 0.05) (Table 1).

**Table 1:** **Correlation between chemerin gene expression in ovaries of PCO rats with insulin**, **glucose**, **HOMA**-**IR index**, **testosterone**, **progesterone and body mass index** (**BMI**) **after PCO induction and treatment of PCO rats with pioglitazone and metformin**

Δ**Chemerin mRNA (after treatment)**		**Chemerin mRNA (PCOS)**		**Variables**
**p**	**r**	**p**	**r**	
0.0847	0.361	0.0327	0.661*	Glucose (mg/dl)
0.0432	0.578*	0.0087	0.754**	Insulin (μg/l)
0.0916	0.291	0.0414	0.523*	Testosterone (ng/ml)
0.0382	0.619*	0.0091	0.827**	Progesterone (ng/ml)
0.0478	0.564*	0.0276	0.678*	HOMA-IR
0.0739	0.378	0.0312	0.654*	BMI

*Correlation is significant at the 0.05 level.

**Correlation is significant at the 0.01 level.

Correlation coefficient (r) and statistical significance (P) are indicated.

**(Δ)** change in chemerin gene expression level after treatment.

## Discussion

PCO is a heterogeneous syndrome characterized by hyperandrogenism, insulin resistance and obesity [24]. The mechanism that is responsible for insulin resistance is unclear and several hypotheses have been suggested. Because obesity is linked to insulin resistance and many women with PCO are obese, it is possible that, at least in a subgroup of patients, insulin resistance is worsened by excessive adipose mass or abnormal secretion or action of adipocytokines [25]. Chemerin is a newly identified adipokine whose systemic levels are elevated in obesity and positively correlate with markers of the metabolic syndrome such as body mass index, triglycerides, high-sensitivity C-reactive protein [15,26]. Recently Tan et al demonstrated an increase of serum and subcutaneous and omental adipose tissue chemerin expression in women with PCO [10]. Chemerin is also expressed in ovary of animal and human, but few data exists on regulation of chemerin gene expression in polycystic ovaries [14,27]. Here we present novel data showing an increase of chemerin gene expression in rat ovary after induction of PCO. The animals in PCO group gained more weight, had higher plasma insulin and glucose levels and showed an elevation of HOMA-IR; an index of insulin resistance. Our findings was in agreement with results of Wang et al which has shown the higher level of chemein expression in the ovary of dihydrotestosterone (DHT) induced PCO rats [15]. Several experiments have demonstrated that chemerin may play a role in pathophysiology of PCO in animal or human by direct action on ovary [10,13,14]. We know that formation of polycystic ovaries is critically associated with abnormal steroidogenesis. It has been found that chemerin decreases estradiol secretion and suppressed FSH-induced progesterone and estradiol secretion in preantral follicles and granulosa cells by inhibition of aromatase and p450scc expression [13].

It is also well recognized that development of polycystic ovaries is associated with new blood vessel formation [28]. Although there is no data about the effect of chemerin on ovarian angiogenesis, however it is plausible that chemerin may act as angiogenic factor. Recently Bozauglo et al using an *in vitro* angiogenesis assay has shown that chemerin induced the formation of capillary-like structures, a process which occur in obese patient and result in adipose tissue expansion [12]. These observations lead to the hypothesis that increasing the chemerin gene expression in ovary of PCO rats may alter ovarian steroidogenesis or angiogenesis and may play a role in the development and progression of this reproductive disorder.

It has been demonstrated that alteration of metabolic and endocrine function of adipocytes result in a higher secretion of proinflammatory substances including tumor necrosis factor alpha (TNF-α) and interleukin-6 (IL-6) [29]. Many reports have demonstrated that these potent inflammatory compounds alter normal physiology of ovary such as steroidogenesis and enhance the risk of PCO development [17,30]. Presence a positive association between increasing body weight and ovarian chemerin gene expression in this study raises the possibility that alteration of chemerin gene expression in ovary of PCO animals may be, in some part, due to increasing fat mass and releasing higher inflammatory mediators such as IL-6 and TNF-α. This hypothesis is supported by the fact that TNF-α increases adipocyte chemerin and elevation of serum TNF-α leads to higher systemic chemerin in mice [31].

We also found the higher plasma insulin level in animals with PCO compared with healthy rats and its positive association with ovarian chemerin gene expression. Interestingly, insulin elevates chemerin in human adipose tissue explants *in vitro*, and systemic chemerin increases after prolonged hyperinsulinemia in healthy individuals. This finding suggests that hyperinsulinemia which occur in our experiment may enhance the chemerin gene expression in polycystic ovaries and it may have role in progression of impaired steroid production [32].

Consistent with previous results, our study showed that chemerin has role in PCO development and manipulation of chemerin gene expression or its signaling may be a novel therapeutic approaches in the treatment of PCO patients. To clarify this hypothesis we test the effect of pioglitazone and metformin on the ovarian chemerin gene expression in normal and PCO animals. To our knowledge, this is the first report demonstrating that treatment of PCO rat with pioglitazone and metformin alter chemerin gene expression in the ovary. Here we reported for the first time that 21 days treatment of PCO rats with metformin and pioglitazone reduced ovarian chemerin mRNA and protein abundance with a concomitant decrease in insulin resistance in PCO animals. Reduction of chemerin gene expression was more pronounced in PCO rats in the presence of pioglitazone compared with metformin, while these drugs had no effects on the basal levels of ovarian chemerin mRNA and protein in healthy animals. We also found improvement of insulin resistance in animals treated with pioglitazone or metformin. Animals in these groups showed lower plasma insulin and glucose level and HOMA-IR compared with PCO animals.

Pioglitazone and metformin are the most important insulin-sensitizing agents currently used most often in clinical practice to improve insulin resistance of PCO patients via different mechanisms which are not thoroughly understood [33,34]. A diverse beneficial effect of pioglitazone and metformin on the treatment of PCO has been described in recent years [35]. Alteration of numerous genes involved in different ovarian functions such as cell proliferation, steroid production and new blood formation by these drugs have been detected and it demonstrate that their positive effects on ovarian function in PCO patients may be multifactorial [35,36]. Recently it has been found that troglitazone or metformin reduce the secretion of chemerin from adipoe tissue and metformin can also reduces chemerin blood levels in concomitant with improving insulin sensivity and decreasing BMI in women with PCO [10]. It has also been demonstrated that both drugs have anti-angiogenic actions, suppresses ovarian androgen production and reduce the secretion of proinflammatory factors including IL-6 and TNF-α [37-39]. Given our findings and those from other groups we hypothesized that these drugs may improve functional and endocrine disturbances of polycystic ovaries, in some part, by suppression of ovarian chemerin gene expression and attenuation of its adverse effect on normal functions of polycystic ovaries.

## Conclusion

In conclusion, using a murine model of PCO, we provided novel evidence that chemerin is an important adipocytokine which may contribute to the dysregulation of ovarian function in PCO. Our study indicated that ovarian chemerin gene expression is associated with several key parameters of the metabolic syndrome in PCO status. Oral administration of pioglitazone and metformin to PCO rats altered the ovarian expression of chemerin genes. These results suggest that some therapeutic effects of metformin and pioglitazone in PCO may be due to their direct actions on ovarian chemerin gene expression.

## Competing interests

The authors declare that they have no competing interests.

## Authors’ contributions

SRFT and MRT were the supervisors and designed the study. MRT carried out the molecular studies. SRFT carried out the hormone assays. All authors read and approved the final manuscript.
